# Can the Development of Orphan Drugs Include Wider Patient Engagement? A Citizens' Jury to Explore a Promissory Notion

**DOI:** 10.1111/hex.70524

**Published:** 2025-12-05

**Authors:** Julia Frost, Widening Engagement Patient Advisory Group, Jessica Mandizha, Chantal van den Dungen, Lauren Asare, Catherine Pope

**Affiliations:** ^1^ Health and Community Sciences, South Cloisters University of Exeter Medical School, University of Exeter Exeter UK; ^2^ Royal Devon University Healthcare NHS Foundation Trust, G18, Bowmoor House, Royal Devon and Exeter Hospital (Wonford) Exeter UK; ^3^ ABFFP Association Belge Francophone contre La Fibrose Pulmoniare Avenue Cantelaube Rebecq Belgium; ^4^ Patient and Public Involvement, NIHR Applied Research Collaboration South West Peninsula (PenARC) University of Exeter Exeter UK; ^5^ Nuffield Department of Primary Care Health Sciences Oxford UK

**Keywords:** deliberative democracy, drug development, imaginaries, patient engagement, promissory, orphan drugs

## Abstract

**Background:**

Drug development practices for rare diseases promote a *promissory* notion that the drug pipeline will succeed (with the next new drug/drug trial) and an *imaginary* of widening patient engagement (often desired but yet to be enacted). Contemporary industry practices of patient engagement are shaped by this promissory, and engagement often includes limited patient perspectives, typically relying on patients from established Patient Organisations or identified by Contract Research Organisations.

**Objective:**

We sought to explore whether more deliberative methods might enable wider, more diverse patient engagement for orphan drug trials.

**Design:**

Citizen's Jury co‐designed with patient advisors.

**Setting and Participants:**

Results of an earlier ethnography of a biotech company's patient engagement practices, along with findings from clinical trials for patients with idiopathic pulmonary fibrosis, clinical practice and patient testimony, were presented to the Citizens' Jury made up of patients and carers.

**Results:**

Jurors discussed and offered a ‘verdict’ on trial materials and processes that would optimise the engagement of more diverse patients for drug trials for rare diseases, suggesting that more could be done at an organisational level to ensure that potential trial participants were able to ‘engage’. They asserted that the industry could do more to understand the unmet needs and wishes of a wider group of patients and should seek input from more diverse groups.

**Discussion:**

The Citizens' Jury called for practices to enable wider engagement—for both drug trials and drug trial design—and more transparency about the risks associated with engagement, for individual patients and currently marginalised groups.

**Conclusions:**

Current drug development practices reify expert patient perspectives and often ignore the views of the wider group of patients who may participate in trials and/or use new medicines developed. More deliberative methods of engagement have the potential to democratise drug development and ensure that new medicines and trials meet the needs of a broader patient demographic.

**Patient or Public Contribution:**

A patient advisory group (PAG) comprising six people with IPF gave input into all aspects of the research design and conduct, including co‐design of the Citizens' Jury. Two patients from international patient organisations served as a steering group (SG). Members of both groups provided their interpretations of the study findings and gave insight into their experiences in clinical design and participation.

## 1 Introduction

Orphan drugs are medicinal products for the diagnosis, prevention or treatment of life‐limiting but relatively ‘rare’ or orphan diseases. It is claimed that orphan drugs are not developed by the pharmaceutical industry for economic reasons but in response to unmet needs [1]. However, the international orphan drug market is predicted to be worth about $400 billion by 2030 [2], with patient engagement (e.g., in setting up patient registries) increasingly viewed as ‘adding value’ to this enterprise [3].

By definition, science and technology are future‐oriented businesses that produce ‘visions’ and ‘promises’ to substantiate their activities (Borup et al. 2006). As such, this ‘promissory science’ centres on emergent, sometimes yet to be realised, technologies (e.g., personalised medicines) [4]. International industry regulators require that clinical trials are ‘conducted with authorised medicinal products and on patients with the same characteristics as those covered by the authorised indication’ [5] and suggest that pharmaceutical companies work with Patient Organisations, where ‘more educated and articulate patients’ are able to input into the drug development life cycle [6]. However, patients with rare diseases are significantly under‐represented in stakeholder groups and surveys about drug development, and participants in clinical trials often do not reflect the diverse populations for which healthcare interventions are needed [7]. For now, wider patient engagement is ‘imaginary’, by which we mean it is desired but not (yet) realised [8]. The current practice of inviting some ‘expert’ patients ‘to have a seat at the table’ risks further advantaging those who are able to engage, while disadvantaging those who cannot [9, 10].

Critics have argued that democratic knowledge production should not privilege a few experts (so‐called ‘epistocracies’) but should instead include people with everyday experience and diverse perspectives [11]. Pharmaceutical companies, Patient Organisations and expert patient alliances dedicated to a rare disease or group of rare diseases may be ‘epistocracies’ or ‘knowledge rulers’ but cannot represent all patients who have a condition [12]. Indeed, *all* patients are in some way competent ‘expert by experience’ [13].

## 2 The Widening Engagement Project

The overarching objective of the Widening Engagement Project (funded by a UKRI/MRC innovation scholarship) was to strengthen knowledge exchange between academia and the biomedical industry. A respiratory physician told us that, because of the challenges associated with the delivery of care for patients with idiopathic pulmonary fibrosis (IPF—a progressive degenerative lung disease) in the United Kingdom (e.g., the limited number of drugs licenced to effectively treat symptoms, which need to be prescribed by specialist physicians [14, 15]), and issues of age, geography, poverty and transport [16], some patients struggle to access clinical trials, which is often the only way that they can access new drugs that are in the development phase and pre‐licence (Michaeli et al. 2023). At the same time, a small biotech company (hereafter ‘Biotech1’) were developing a new medicine for IPF and agreed to the secondment of J.F. as an embedded researcher [17, 18], to identify opportunities where patient engagement policies and practices could be extended to engage a wider group of patients in the drug development pipeline. Therefore, as a group of patients, clinicians and academics, by widening patient engagement, we mean purposeful initiatives to improve opportunities to engage in clinical trial design and access clinical trials for a more diverse and inclusive group of patients and carers.

To inform all aspects of the research design and delivery, steering and advisory groups were convened and met regularly. The steering group (SG) comprised members of expert patient organisations and health professionals and a medical sociologist (C.v.d.D., J.M. and C.P.). The patient advisory group (PAG) consisted of six people who have IPF or who care for someone with IPF, who may or may not have participated in patient support groups and who may or may not have participated in clinical trials of new medicines for IPF (Widening Engagement Patient Advisory Group).

For the wider project, firstly, we conducted a literature review to identify how patients and the public engage with drug development for rare diseases [19]. Secondly, we produced an ethnography of our industry partners' engagement practices (in progress). Thirdly, we captured the clinical trial experiences of local IPF patients and juxtaposed them with industry‐commissioned reports of IPF patients' experience [20]. Finally, we co‐designed an accessible and creative form of engaging with IPF ‘publics’ [21] to ensure that any recommendations made spoke to a range of stakeholders [22]. The aim of the final phase of this project was to work with patients to see whether more deliberative methods of engagement might enable the imaginary of widening patient engagement for orphan drug trials to be realised, and it is this part of the project that we focus on in this paper.

We worked with the definition of deliberative democracy ‘as any practice of democracy that gives deliberation a certain place’ [23, p. 2]. Deliberative theory has the potential to help us think about patient engagement in drug development, as it makes a distinction between participatory democracy, which is consultative, and deliberative democracy, which is discursive and dialogic [24]. Within healthcare, there have been several large‐scale attempts to instigate democratic principles of patient engagement in decision‐making [25], with several UK examples noteworthy: Firstly, NICE convened a Citizens Council to capture the informed views of the public in shaping the institute's value judgements, with one report specifically about drug pricing for rare diseases [26]. Secondly, the National Health Service Citizen (NHSC) established a process of meta‐deliberation designed to capture citizen perspectives to shape the system itself; however, many participants found the forms of deliberation undemocratic [27]. More recently, the Nuffield Council of Bioethics has undertaken a Citizens' Jury to explore public views on assisted dying in England, as a means of applying deliberative principles to ‘a highly complex, sensitive, and ethically‐charged topic’ [28, p. 11].

## 3 Methods

Citizens' Juries were developed by the Jefferson Centre (now Centre for New Democratic Processes) in the 1970s, who produced a trademarked set of components ‘to provide citizens the opportunity to learn about an issue, deliberate together with a diverse group of their peers, and develop well‐informed solutions to challenging public issues’ [29]. Within health research, Citizens' Juries are often described as having been adapted (e.g., over 1 or 2 days rather than the 4–5 proscribed by the Jefferson Centre), although typically the rationale and impact of any adaptation are not provided [30]. However, Friske et al. (2019) contend that ethical practices (e.g., recognising and responding to individuals and groups who have historically been disadvantaged and who remain vulnerable when engaged in the research space) must be the drivers of an inclusive citizen science to meet the needs of populations currently underserved by medical research.

### 3.1 Design

We co‐designed a Citizens' Jury to bring together a range of people interested in optimising clinical trials for people with IPF. The PAG recommended who should be invited to provide witness testimony (e.g., a range of people who patients might encounter in their journey along the IPF patient pathway) and chose to provide their witness testimony by film because they were concerned that they would not be well enough to attend in person; however, one of the four members of the PAG chose not to provide a testimony, and their wishes to do so, without justification, were respected. In keeping with the spirit of knowledge exchange, two members of staff from Biotech1, to which J,F. was seconded, observed the Citizens' Jury as a form of organisational learning (e.g., the processes of the jury). The jury was asked to discuss and input into the question: ‘To expand orphan drug engagement to widen their reach and benefit, can we co‐design accessible resources for patients, and organisations that recruit patients to orphan drug trials?’ They were provided with witness testimony related to the question and provided with an opportunity to discuss and agree on issues that were important to them and which could inform recommendations [29] (Table 1).

**Table 1:** Schedule of events for the Citizens' Jury.

Time	Activity	
Before the day	Please read the Patient Recruitment Materials that you have been sent with your information pack	All participants
10.00–10.20	Arrival, refreshments, welcome and introductions	Julia Frost
10.20–10.40	Introduction to Citizens' Jury and deliberation	Lauren Asare
10.40–11.00	Icebreaker activity to allow jury members and facilitators to get to know each other	Catherine Pope
11.00–11.30	Witness testimony: Access to specialist services, clinical trials and impact	Jessica Mandizha
11.30–12.00	Improving the quality of life and supporting patients	Steve Jones
12.00–12.30	Current and future clinical trials	Phil Molyneux
12.30–13.00	Widening patient engagement	Widening Engagement Patient Advisory Group
13.00–13.30	Co‐designed patient resources	Julia Frost
13.30–14.00	Lunch with witnesses and jurors	All participants
14.00–15.00	Jury deliberations: Table discussions and deliberation with speakers	Julia Frost, Lauren Asare and Catherine Pope
15.00–16.00	Jury deliberations: Feedback from table discussions to the whole room to identify levers for widening patient engagement in drug development What are the implications of what we've heard for practice and policy recommendations?	Julia Frost, Lauren Asare and Catherine Pope
16.00	Thank you and close	All participants

Citizens' Juries typically bring together a randomly selected group of people who have characteristics that are representative of their community [30], but for this project, we only invited people with IPF, or carers of people with IPF, from ‘publics’ which we could identify and access (e.g., via NHS partners and other professional and research networks). Jurors were recruited in several ways to ensure that people with a range of IPF treatment and trial experiences were included. Firstly, all patient participants interviewed in an earlier phase of the research were invited [20]. These were people who have IPF, who tend to live in a rural or semi‐rural location and who attend a specialist interstitial lung disease (ILD) centre in the South West of England, and who may or may not have participated in one or more clinical trials. Their perspective was important, as our earlier research indicated that they experienced difficulty in securing an IPF diagnosis and often had to travel to a regional centre for clinical review, prescription of specialist medicines and access to clinical trials of new medicines. Secondly, people who have IPF and who live in close proximity to a large city were more ethnically diverse, and those who attend a specialist ILD centre in the South East of England were invited by their ILD Specialist Nurse. Finally, members of a support group for people with pulmonary fibrosis in the South West of England were invited by members of the research team at one of the support meetings; this third group had a mixture of the above attributes, but also experience of self‐organisation into a support group. Having been approached and provided with information about the purpose of the jury via their clinicians and peers, all jurors were recruited directly by J.F.

As members of these groups were approached in a range of ways, a definitive number of those approached is not known, although we worked towards the recommendation that there should be 12–24 jurors for a Citizens' Jury to work effectively [29]. A Citizens' Jury usually meets over several days [29], but understanding that a longer process would be a burden to these participants, this jury met on a single day. To be inclusive, jurors were able to select attendance in person (with a family member or friend to support them), by videoconferencing, or by telephone. The PAG told us how important these options were for someone with a terminal lung disease, who may be on oxygen therapy and/or experiencing fatigue. Furthermore, during the earlier phases of the project, some IPF patients had told us that they avoid patient support groups because seeing patients who were at a later disease stage than themselves, as they found this upsetting [20]; therefore, virtual attendance offered a degree of distance and anonymisation.

Members of the PAG suggested that we conduct the Citizens' Jury in an accessible location, which people with IPF could drive to, if they wished to avoid public transport (as they have a respiratory condition, and many had been shielding due to the recent Covid pandemic). Members of the SG advised us to provide places for IPF patients to rest and provide electronic sockets for people who require oxygen therapy. Thus, the Citizens' Jury was conducted in a hotel, where jurors and their carers could stay overnight and rest when required.

Witnesses were chosen by the research team (Table 1) to provide a range of lay, clinical and academic perspectives about clinical trials of new medicines for IPF and clinical trial design. They were briefed to provide input that would be digestible, easily understandable, simple and clear and use language that a non‐specialist audience would understand. Jurors were given the opportunity to cross‐examine the witnesses before deliberating on their verdict.

To maintain a relaxed environment, we avoided video or audio recording and instead recorded field notes and collated the materials that the jurors generated (e.g., their notes made on flipchart paper). However, with prior consent, the deliberative process was ‘recorded’ by an illustrator.

J.F. and C.P. collected and synthesised the flipcharts and handwritten notes and checked their recordings of quotes for accuracy. Their content and synthesis were then agreed upon by all authors. We used a simple form of sense‐making (or ‘coding’) that involved noting patterns in the process and content of the deliberations [31], which aligned with the patient pathway for accessing clinical trials of an orphan drug [32]. Jurors evaluated the process by completing a semi‐structured and self‐administered questionnaire in the days after the Citizens' Jury [33] (Supporting Material).

## 4 Results

In June 2024, 19 people attended this Citizens' Jury as jurors at an accessible conference centre in Greater London. Of these, 11 were people with IPF, and 8 were carers or relatives of those people. 17 chose to participate in person, and 2 joined via Zoom (Table 2).

**Table 2:** Public representation in Citizens' Jury.

Recruitment group	Patients	Carers
Interviewees from Phase 1 of research—South West	3 (all face to face)	3
IPF patients from South East	4 (3 face to face and 1 online)	2
Members of the support group—South West	4 (3 face to face and 1 online)	3
	*n* = 11	*n* = 8

**Figure 1:**
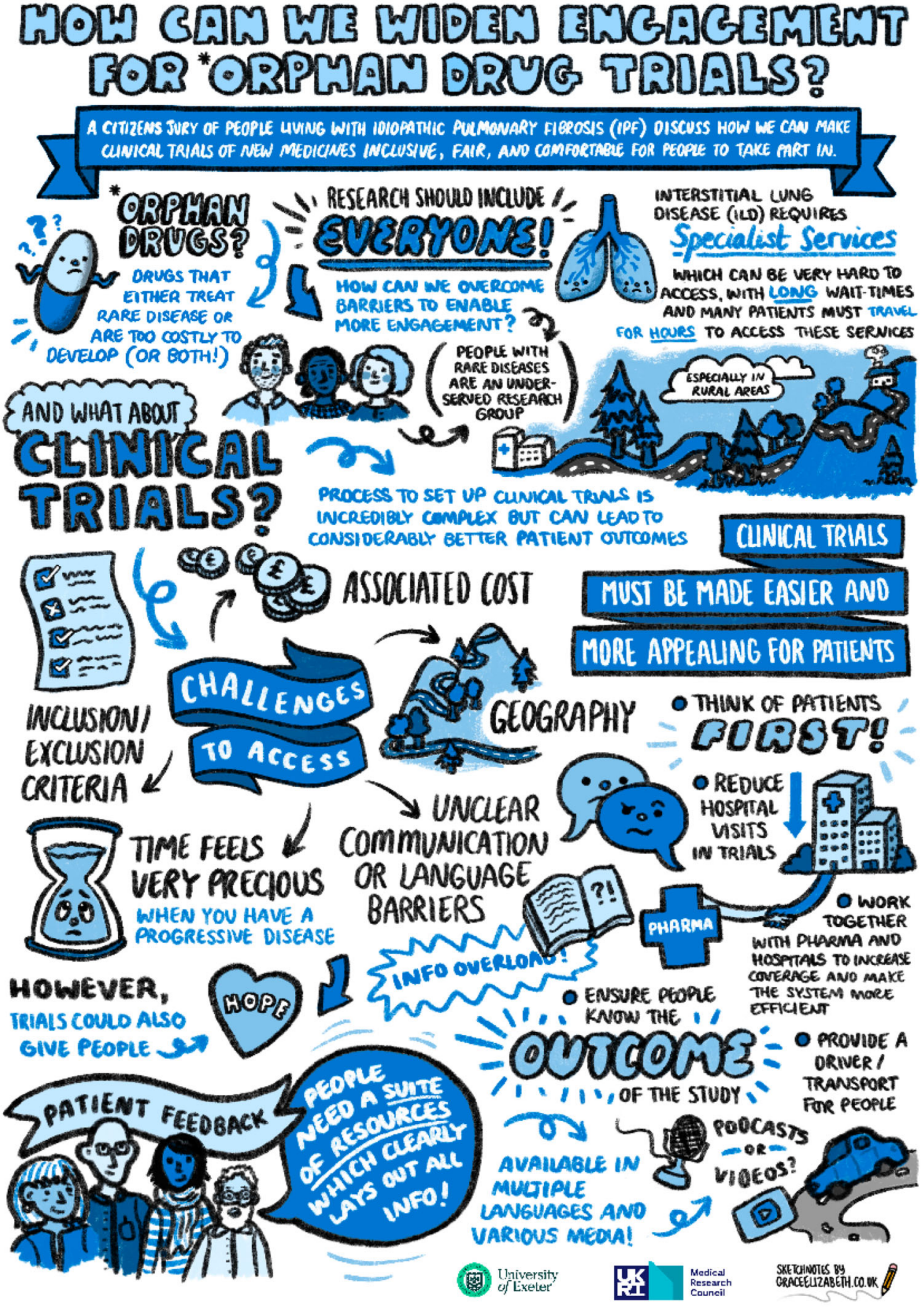
Infographic of the deliberative process and outcomes.

While the deliberations were taking place, the process was recorded by an illustrator https://www.graceelizabeth.co.uk/ both to protect the identities of the jurors and to provide a visual representation of the questions and challenges that the jurors were discussing and seeking to address (Figure 1).

## 5 Diagnosis and Information About IPF

Jurors proposed that when people are diagnosed with IPF, they should be given sufficient information about the disease before they are provided with information about clinical trials. Sometimes these two processes are conflated when patients are referred to tertiary centres for diagnosis, which are where clinical trials of new medicines are conducted. They suggested that bringing a relative or friend with them to clinical appointments is valued, as there is a considerable amount of information to take in.

People need to know about the disease before they know about trials.

Juror 1

They recommend that people be given more information about the different organisations involved in their care (e.g., definitions and explanations of primary/community care, secondary/local hospital care, and tertiary/specialist care), and they want the relationships between organisations to be easier to understand, with better communication between them. They want these various organisations to be both informed about and provided with clear information about IPF.

Currently patients have different experiences between large and small hospitals, and need clarification about the roles of GPs, local and specialist care and, how to improve communication between them.

Deliberative Group 1

Jurors proposed that a health professional needs to talk to people with IPF about their various drugs and treatments, to make consultations more positive and holistic. People with IPF also need to better understand what their test results mean, to inform their understanding and decision‐making. Where referral to peer support is provided (e.g., to Action for Pulmonary Fibrosis), this is valued and recommended.

Patients need a range of support options that are peer or professional support at a local and national level.

Deliberative Group 2

### 5.1 Trial Information

Jurors made recommendations about the language used in materials used to recruit people with IPF to trials. They want the language to be simple (e.g., use of bullet points) and positive. The use of section headings and colour could aid navigation. They want more information about inclusion and exclusion criteria (e.g., why they are being approached to participate), and they want to clearly understand any risks associated with participation.

Counter to the current templates provided by international regulators, jurors made specific recommendations for the structure of Participant Information Sheets, including: an initial Summary of the study in simple language, with any tables or technical information provided in an annexe. They want to see the Schedule of Events clearly translated into words, so that a participant can understand exactly what will happen at every stage of the trial (e.g., what they are required to do, for how long, and why). Information is required about reimbursement of time and whether this is extended to other costs (e.g., for their travel and for their carer).

Information sheets need a summary at the beginning. The data protection section is currently really wordy, and perhaps that can go in an annexe? We also discussed the use of language, different fonts and flowcharts.

Deliberative Group 5

Jurors also proposed that paper‐based information be supplemented with clear explanations of technical processes and opportunities to ask questions. They recommended a suite of resources that can be tailored to individual needs: posters (e.g., representing people from different cultural and socio‐economic backgrounds), short videos, podcasts, webinars and peer support. Information must also be available for their friends and family to understand what a trial will entail.

We talked about advertising to “people like me”—pictures of people from ethnic groups, in local papers or on hospital posters.

Deliberative Group 4

Jurors recommended that clinical staff provide more information about where the person with IPF is in relation to clinical trials: Individually, in terms of waiting to hear about a suitable trial or if they are not suitable for a trial. Collectively, in terms of what is happening in the current landscape of trials for IPF, both locally and internationally.

Feedback is vital—don't leave people in the dark. I agreed to join a trial, but I just kept getting nothing back.

Juror 11

### 5.2 Trial Processes

Jurors recommended that resources and arrangements are required to enable people with IPF to access clinical trials. This depends on individual need but could include transportation: not just from home to the hospital car park, but also around the clinical site. While there was agreement that costs need to be reimbursed for both patients and carers, there was less agreement about paying participants to take part in a trial, although it was acknowledged that this would be appropriate for people who would otherwise struggle to take part.

How to access a trial: physically get to the hospital and be offered a driver. Not just for healthy people, but also if they use a wheelchair and how they get from the carpark to the clinic.

Deliberative Group 3

They suggested that more needs to be done to make patient‐reported outcome measures more accessible. This could be achieved by making them audible or by having someone come to the patient's home to assist them with completing the questionnaire.

You just need to make them more accessible!

Juror 4

Jurors recommended that patients participating in a trial and/or having completed a trial should receive feedback about the trial progress and/or results. It was emphasised that it is frustrating to take part in a trial and never be informed what that participation has contributed towards.

I participated, but was given no feedback, I was contacted by a study and at end asked to give the equipment back.

Juror 10

### 5.3 Engagement in Clinical Trial Design

Jurors reported that events like the Citizens' Jury would be useful for patients to input into how clinical trials are *designed*. Being group‐based, face‐to‐face and ‘live’ brings a social aspect to participation. Jurors agreed that engaging with clinical trials is difficult to navigate. They emphasised that the onus must be on pharmaceutical companies to make it easier for patients from a range of backgrounds (e.g., who are not in patient organisations or for whom English is not their first language) to participate. This will require companies to go to where these patients are.

They need us to help them identify barriers to participation and the risks of participation.

Deliberative Group 5

### 5.4 Feedback About the Citizens' Jury Process

Following the Citizens' Jury, the jurors provided feedback on the process (Electronic supporting material). Most jurors either strongly agreed or agreed that they were adequately prepared for the Citizens' Jury and understood what it would entail and able to share their ideas, opinions and experiences. However, this was more difficult for the two jurors who joined the Citizens' Jury online and where technological challenges inhibited their effective deliberation with the jurors in the room. Few jurors recalled seeing or looking at the videos about Citizens' Juries that were embedded in the briefing document [links to these videos to be included here], and few agreed that they had changed their mind as a result of taking part in deliberations. However, most jurors strongly agreed that in the future they would participate in a trial, if and when one became available, and that they would share any study information with carers and family members.

## 6 Discussion

It was evident from the quality of deliberations and dialogue around and between each table in our jury that individuals recruited from different clinical and geographical environments had different experiences of clinical trials of orphan drugs. However, our diverse jurors were able to articulate and agree on a set of recommendations that they considered would enable a wider group of patients to be engaged in orphan drug trial design and conduct. They proposed that more information about both diagnosis and usual care needed to be foregrounded, to enable patients to make informed decisions about trial participation ([34, 35]; van Rossenburg et al. 2021). Importantly, the jury suggested that not only do standard templates for patient information need to be re‐structured and simplified [36, 37], but that this needs to be supplemented with more bite size information packaged to reflect the wider demographic of patients who could participate [7], with more time allocated for health professionals to tailor the information to patients' individual needs [38]. These findings align with assertions that the public can and should have a role in making science more socially accountable and ethical [39] and with proposals that the pharmaceutical industry, and science more generally, needs greater accountability to the general public, rather than only seeking to work with expert citizens [40].

Like other Citizens' Juries who have engaged underserved populations, we convened a jury that was descriptively representative of the population of IPF patients (e.g., in terms of age, gender, ethnicity, location, disease severity, and models of treatment and trial delivery) rather than a random sample [30, 41, 42]. On the advice of the PAG, we opted to hold the jury in a central location, where patients with respiratory conditions could drive or be driven to without fear of being infected on public transport, and because travelling was physically draining for many of the participants, they also required accommodation for themselves and their families [author publication]. In addition, the PAG recommended that we provide patients with both face‐to‐face and online options for participation, as some participants may be too unwell to travel or may not want to see how unwell IPF patients can become [43]. A limitation of this study is that hybrid conferencing facilities were limited, which meant that the two hybrid participants were only able to deliberate with each other, or with the rest of the jury via a facilitator (L.A.). If we were to conduct a further hybrid Citizens' Jury, we would ensure that truly accessible means of deliberation are provided [44]. We used arts‐based methods of engagement, including the use of video testimony and illustration (Kashefi et al. 2004), which the jurors reported worked well to engage them and convey their strength of feeling; however, a risk of ‘upstream public engagement’ is an inability to demonstrate any ‘downstream impact’ (Gooberman Hill et al. 2008; Greenhalgh et al. 2016). Outputs from this jury have been shared with policymakers and industry representatives; however, any mechanisms to hold them to account for actioning these recommendations are currently missing [45].

With the fast pace of industrial medicine [46] and the reactive nature of pharmaceutical engagement (Polock et al. 2010; Duggal et al. 2020), industry currently chooses to engage public representatives whose views are more likely to align with their own ([11]; Rabaharisoa et al. 214a; [8]). The dominant promissory narrative (that the drug pipeline will succeed) downplays the risks inherent in scientific discovery and fails to see that the ‘past is littered with failed futures’ (Brown et al. 2003). Critics suggest that other perspectives are needed (Khushf 2013), as well as more scrutiny of whose interests new technologies or science serve [45]. In this context, the experiential knowledge held by patients can act as a corrective form of governance that has the potential to hold to account the ‘credentialed knowledge’ held by ‘experts’ ([47]; Scheinerman 2023). For example, many IPF patients have different views on pharmaceutical companies and regulators about desired outcomes and how to measure these; they often want new treatments to enable them to undertake simple daily tasks.

We employed one form of deliberative democracy to try to tip the balance of power in favour of a wider, more diverse group of patients than those who are usually invited to have a say [24]. However, a more radical approach to deliberation would be for the pharmaceutical industry to grasp the importance of equality, diversity and inclusivity and address the current power differential between pharmaceutical organisations and their potential end users ([48]; Schneiderhan et al. 2018; [9]). This would enable currently marginalised voices to challenge current drug development processes that construct narrow visions of both patients and engagement ([24, 49]; Jovanoski et al. 2021). Deliberative methods have the potential to replace the current imaginary of patient engagement and promissory of orphan drug development with more meaningful, wider engagement to deliver better treatment options.

## 7 Conclusion

This Citizens' Jury was able to offer a ‘verdict’ on trial materials and processes that could optimise the engagement of a wider group of patients for orphan drug trials. Our jurors emphasised that more could be done at an organisational level to ensure that potential trial participants were able to ‘engage’ with drug development. They asserted that industry could do more to understand the unmet needs of a wider, more diverse group of patients than those with whom they currently engage. A Citizens' Jury is a viable method of engaging with a more diverse group of patients (and their carers) and has the potential to increase the value of patient engagement. The Citizens' Jury we report here called for practices to enable wider engagement in drug trials and drug trial design, and more transparency about the risks associated with engagement, for individual patients and currently marginalised groups. The current epistocracy favours a select group of expert patients, but a wider group of patients will be involved in trialling and using new medicines. More deliberative methods of engagement have the potential to democratise drug development and meet the needs of this broader demographic.

## Author Contributions

**Julia Frost:** conceptualisation, methodology, software, data curation, formal analysis, validation, investigation, funding acquisition, visualisation, project administration, writing – original draft, writing – review and editing. **Jessica Mandizha:** conceptualisation, funding acquisition, data curation, supervision, validation, writing – review and editing. **Chantal van den Dungen:** investigation, writing – reviewing and editing, supervision, data curation, formal analysis. **Lauren Asare:** project administration, resources, writing – review and editing. **Catherine Pope:** conceptualisation, data curation, formal analysis, supervision, writing – review and editing, funding acquisition, validation, investigation.

## Ethics Statement

London—Riverside Research Ethics Committee (REC reference: 22/LO/0186; IRAS project ID: 304904).

## Consent

All participants gave their written consent for the interview in which they participated to be recorded and declared that they understood that the interview would be typed up (transcribed), with all of the information anonymised.

## Conflicts of Interest

The authors declare no conflicts of interest.

## Glossary

**Citizens' Jury** provides members of the public with the opportunity to learn about an issue, deliberate together with a diverse group of their peers, and develop solutions together.

**Deliberative democracy** is a form of discursive democracy in which deliberation is central to decision‐making.

**Idiopathic pulmonary fibrosis (IPF)** is a progressive and degenerative disease that causes scarring (fibrosis) and stiffness of the lungs and makes it difficult to breathe.

**Imaginary** is a desirable form of democracy (e.g., patient engagement) but not yet a necessity.

**Orphan disease** is a rare disease whose rarity results in little or no funding or research for treatments, without financial incentives from governments or other agencies.

**Orphan drug** is a medication targeting orphan diseases.

**Promissory** is an emergent technology (e.g., personalised medicine) that exists in the speculations of its supporters.

**Patient engagement** includes involvement of the patient/members of the public in the design and conduct of the study, in discussion of findings and advice about implications/dissemination (e.g., purposeful initiatives to improve opportunities to engage in clinical trial design and access clinical trials for a more diverse and inclusive group of patients and carers).

## Supporting information

Electronic supplementary material.

## Data Availability

The data that support the findings of this study are available on request from the corresponding author. The data are not publicly available due to privacy or ethical restrictions.
