# Supplementary material for: Can the Development of Orphan Drugs Include Wider Patient Engagement? A Citizens' Jury to Explore a Promissory Notion
Source: Health Expect. 2025 Dec 5;28(6):e70524. doi: 10.1111/hex.70524 (PMC12679173; doi:10.1111/hex.70524)
Supplement: Supplementary file 1 — Electronic supplementary material. [file HEX-28-e70524-s001.docx]

**Supplementary material: Widening Patient Engagement for Orphan Drug Trials – Citizens’ Jury Evaluation**

**N=19** (* indicates online comments)

1. The Jury’s task and purpose were made clear to me:

|  | Strongly agree | Agree | Not sure | Disagree | Strongly disagree | Missing data |
| --- | --- | --- | --- | --- | --- | --- |
| In room | 9 | 4 |  |  |  | 4 |
| Online |  | 2 |  |  |  |  |

*Comments:*

It took me a while to grasp

* There was some problems connecting online which made it difficult to hear most of the introduction parts, so I’m not sure I understood the purpose initially.

1. I had adequate information and resources to support my participation:

|  | Strongly agree | Agree | Not sure | Disagree | Strongly disagree | Missing data |
| --- | --- | --- | --- | --- | --- | --- |
| In room | 7 | 8 |  |  |  | 2 |
| Online | 1 |  |  | 1 |  |  |

*Comments:*

*Tech issues, couldn’t hear most of the conversation from one presenter in particular (stood away from mic) If the text could be in bullet points rather than chunks of text this would be better

*As above, the technical side meant it was difficult to follow along

1. The speakers provided me with a broad range of perspectives:

|  | Strongly agree | Agree | Not sure | Disagree | Strongly disagree | Missing data |
| --- | --- | --- | --- | --- | --- | --- |
| In room | 11 | 3 |  |  |  | 3 |
| Online | 1 | 1 |  |  |  |  |

*Comments:*

The patient advisory group video provided more patient relatability alongside academia

Very interesting

*There was a good mix of speakers, all of which were really useful

* It was useful to hear from a range of speakers and I will look forward to looking back at the slides to catch up on the details that I missed

1. The small group discussions enhanced my participation and understanding:

|  | Strongly agree | Agree | Not sure | Disagree | Strongly disagree | Missing data |
| --- | --- | --- | --- | --- | --- | --- |
| In room | 11 | 4 |  |  |  | 2 |
| Online |  |  |  | 2 |  |  |

*Comments:*

*As there were only two of us online it made it difficult to find out about many more views. It would have been nice to hear from the other attendees in person

* I enjoyed speaking to the other online attendee but is difficult to say that It made a difference to my understanding as there was only two of us.

1. There was enough time for discussion and debate and for everyone to have their say:

|  | Strongly agree | Agree | Not sure | Disagree | Strongly disagree | Missing data |
| --- | --- | --- | --- | --- | --- | --- |
| In room | 5 | 10 |  |  |  | 2 |
| Online |  | 1 | 1 |  |  |  |

*Comments:*

Could have been longer!

There is never enough time for discussion in full

Interesting

*I felt there was adequate time talking with my partner online but I don’t know how this worked in the room

*As there were only two of us online it made it difficult to find out about many more views. It would have been nice to hear from the other attendees in person

1. I felt comfortable sharing my ideas, opinions and experiences:

|  | Strongly agree | Agree | Not sure | Disagree | Strongly disagree | Missing data |
| --- | --- | --- | --- | --- | --- | --- |
| In room | 10 | 5 |  |  |  | 2 |
| Online | 2 |  |  |  |  |  |

*Comments:*

An excellent space to talk

I hope I didn’t dominate

*Welcoming, safe space

* There was an understanding of trust almost immediately, which made it easy to share even personal details

1. I changed my ideas and opinions through listening to others:

|  | Strongly agree | Agree | Not sure | Disagree | Strongly disagree | Missing data |
| --- | --- | --- | --- | --- | --- | --- |
| In room |  | 4 | 7 | 2 |  | 4 |
| Online |  |  |  | 1 | 1 |  |

*Comments:*

Our group mostly agreed

I agreed with the ideas and the opinions shared

It was explained why you cannot promote trials through overt adverts

Neutral

*Maybe if in the room, or had more discussions with other attendees I might have done

* There was not a lot of differing discussions online, perhaps there would have been more opportunity to do this in the room

1. I felt my voice was heard and my views contributed to the recommendations:

|  | Strongly agree | Agree | Not sure | Disagree | Strongly disagree | Missing data |
| --- | --- | --- | --- | --- | --- | --- |
| In room | 8 | 7 |  |  |  | 2 |
| Online |  | 1 | 1 |  |  |  |

*Comments:*

*I was able to share, but did see how this contributed in person, although was told that a list of our online recommendations was shared with the wider group

* The conversation with [facilitator] made me feel like we were being heard, and I felt confident my thoughts would be passed on although we were recommended not to say for the final discussion time where our views would be shared with the room and so I don’t know whether this made a difference to the overall recommendations

1. Everyone who participated in the Citizens’ Jury had an equal opportunity to share their opinions and ideas:

|  | Strongly agree | Agree | Not sure | Disagree | Strongly disagree | Missing data |
| --- | --- | --- | --- | --- | --- | --- |
| In room | 7 | 8 |  |  |  | 2 |
| Online |  |  |  | 1 | 1 |  |

*Comments:*

*Huge disparity between those in the room and those online

*I was not able to participate in the same way, and share in the same way due to being online

1. Before the event I watched the videos that were included in the programme for the day:

|  | Strongly agree | Agree | Not sure | Disagree | Strongly disagree | Missing data |
| --- | --- | --- | --- | --- | --- | --- |
| In room |  | 1 | 3 | 3 | 7 | 3 |
| Online |  | 1 |  |  | 1 |  |

*Comments:*

I didn’t receive any info, as there was a mix up with my notes

No videos offered to be shared before the event

Did not see a video before the event

None available

There was a mix up and we didn’t get any information

The link didn't work for me

No. The link didn’t work on the train

I didn’t realise that there were videos

Yes. Saw them and they were informative

* I didn’t see any videos sent beforehand

1. Before the event I read the pre-read materials that were sent with the programme:

|  | Strongly agree | Agree | Not sure | Disagree | Strongly disagree | Missing data |
| --- | --- | --- | --- | --- | --- | --- |
| In room | 6 | 3 |  | 2 | 2 | 4 |
| Online | 2 |  |  |  |  |  |

*Comments:*

Very clear info. Very colourful/clear

Yes -but a little confused

Very briefly, as I only knew I was coming the night before

I didn’t receive any info, as there was a mix up with my notes

Sadly not

There was a mix up and we didn’t get any information

1. I intend to talk to family and friends about the information discussed at the Citizens’ Jury:

|  | Strongly agree | Agree | Not sure | Disagree | Strongly disagree | Missing data |
| --- | --- | --- | --- | --- | --- | --- |
| In room | 1 | 11 | 1 |  | 2 | 2 |
| Online |  | 2 |  |  |  |  |

*Comments:*

Will mention it at our group

1. If I was eligible for a clinical trial, I would now be more inclined to participate:

|  | Strongly agree | Agree | Not sure | Disagree | Strongly disagree | Missing data |
| --- | --- | --- | --- | --- | --- | --- |
| In room | 9 | 2 | 2 |  | 1 | 3 |
| Online | 1 | 1 |  |  |  |  |

*Comments:*

I was already inclined

* Already in other trials

No. Partner

1. If the opportunity arose for me to assist in the design of a clinical trial, I would now be more inclined to participate:

|  | Strongly agree | Agree | Not sure | Disagree | Strongly disagree | Missing data |
| --- | --- | --- | --- | --- | --- | --- |
| In room | 9 | 3 | 2 |  |  | 3 |
| Online | 2 |  |  |  |  |  |

*Comments:*

Possibly

Cautious/shy in nature

* I would do anything I can to contribute to the research and help others in my similar situation

* If I have the time, I will continue to volunteer

1. What attracted you to coming along to the Citizens’ Jury?

*Comments:*

Information on IPF

Opportunity for more info on IPF

To find out how to improve my husbands condition

Accompanying ‘patient’ to learn more

To meet with others and share our experiences

Trip to [city]. Meeting other people with IPF. Helping to make a difference

The opportunity to have my views heard and improve trial uptake

Was privileged to be asked and looked forward to the opportunity

To learn what it was about

Very interested in learning about research, like to learn and be informed

Support my husband. Promote research for IPF

Learning about Citizens Jurys and their purpose

Information

To have a say in things

Info from [researcher]

* To help others, to see what is out there and if it is in-line with my expectations, and to learn more about my own circumstances and the work that is taking place.

* I was approached to attend through another meeting, and thought it would be good to experience something different.

1. What did you like most about the event?

*Comments:*

Meeting other patients and carers

Topics covered

Topics covered

The sharing of issues around ‘orphan drugs’. The speakers setting the context and providing valuable information

The talks and group discussion

The round table discussions and the speaker input

Table discussions

Open forum

The whole day. Relaxed atmosphere

The information we were given was easy to understand and was very good. I think everything was very good and enlightening. All the people were friendly.

Meeting all the participants

Inclusion

Everything – all the speakers were excellent and supportive

The participation with the talkers and the Jury

* Learning new information, meeting new people, have a greater understanding of my disease and the clinical trials that may go on to help those yet to be diagnosed.

* It was good to talk to the other online person, and also to one of the speakers. I did enjoy hearing from the different speakers although the sound quality was difficult

1. What could have been better for you?

*Comments:*

Understanding the purpose of the gathering in advance

To make more clearly written, to understand the content

Simpler language

A microphone and roving microphone when talking around the tables

A microphone for speakers. Conference room on the ground floor and bedroom nearer to reception

Loop system microphone. Sound was quiet

Better amplification (my hearing is not good)

Information before the event about travel and accommodation.

Travel information

Travel information

Nothing I can think of

N/A

N/A

Nothing comes to mind

Nothing

* The online technology was the only real disappointment. It would have been nice to meet the audience, as there was no real idea on the size of the attendees.

* The sound quality was really tricky, this would have made a difference to the experience.

1. Do you have any other comments about your experience?

Comments:

Thank you for the opportunity, it was really interesting and valuable.

Thank you for a good and informative day

Thank you

A really informative day

No! just a really informative day – many thanks

Thank you to all. It was very interesting, you all done very well

Enjoyed

Good venue and food

Stayed awake!

* It was interesting to hear that there was a lack of ethnic diversity in the room and in the trials. From someone is in an ethnic minority it would be useful to know how trials might be advertised to others from a range of backgrounds, and perhaps target those from minority groups to increase the diversity in attendance.

* Thank you for inviting me, I would like to do any others that you have planned.
